# Shear-Dependent Platelet Aggregation: Mechanisms and Therapeutic Opportunities

**DOI:** 10.3389/fcvm.2019.00141

**Published:** 2019-09-20

**Authors:** Akshita Rana, Erik Westein, Be'eri Niego, Christoph E. Hagemeyer

**Affiliations:** Nanobiotechnology Laboratory, Australian Centre for Blood Diseases, Central Clinical School, Monash University, Melbourne, VIC, Australia

**Keywords:** platelets, shear, aggregation, thrombosis, VWF

## Abstract

Cardiovascular diseases (CVD) are the number one cause of morbidity and death worldwide. As estimated by the WHO, the global death rate from CVD is 31% wherein, a staggering 85% results from stroke and myocardial infarction. Platelets, one of the key components of thrombi, have been well-investigated over decades for their pivotal role in thrombus development in healthy as well as diseased blood vessels. In hemostasis, when a vascular injury occurs, circulating platelets are arrested at the site of damage, where they are activated and aggregate to form hemostatic thrombi, thus preventing further bleeding. However, in thrombosis, pathological activation of platelets occurs, leading to uncontrolled growth of a thrombus, which in turn can occlude the blood vessel or embolize, causing downstream ischemic events. The molecular processes causing pathological thrombus development are in large similar to the processes controlling physiological thrombus formation. The biggest challenge of anti-thrombotics and anti-platelet therapeutics has been to decouple the pathological platelet response from the physiological one. Currently, marketed anti-platelet drugs are associated with major bleeding complications for this exact reason; they are not effective in targeting pathological thrombi without interfering with normal hemostasis. Recent studies have emphasized the importance of shear forces generated from blood flow, that primarily drive platelet activation and aggregation in thrombosis. Local shear stresses in obstructed blood vessels can be higher by up to two orders of magnitude as compared to healthy vessels. Leveraging abnormal shear forces in the thrombus microenvironment may allow to differentiate between thrombosis and hemostasis and develop shear-selective anti-platelet therapies. In this review, we discuss the influence of shear forces on thrombosis and the underlying mechanisms of shear-induced platelet activation. Later, we summarize the therapeutic approaches to target shear-sensitive platelet activation and pathological thrombus growth, with a particular focus on the shear-sensitive protein von Willebrand Factor (VWF). Inhibition of shear-specific platelet aggregation and targeted drug delivery may prove to be much safer and efficacious approaches over current state-of-the-art antithrombotic drugs in the treatment of cardiovascular diseases.

## Introduction

Since Bizzozero first identified the remarkable contribution of platelets in both hemostasis and thrombosis (1), substantial progress has been made in understanding, diagnosing and treating several platelet-related disorders. With technological advances in platelet research, immense insights were generated into the mechanisms underlying “hemostatic” and “thrombotic” activities of these intriguing blood cells.

Platelets, or thrombocytes (2–4 μm in greatest diameter), are anucleate cells produced by megakaryocytes, that circulate in the human bloodstream for 7–10 days before being eliminated by the liver and spleen (2, 3). Primarily, platelets sense and respond to vascular injury by preventing blood loss following vessel damage and tissue trauma. But in addition to this essential hemostatic function, platelets also play a key pathological role in cardiovascular diseases.

Several decades of research into platelets and their varying pro-thrombotic roles at sites of vascular injury have established the synergistic interplay between two distinct, yet complimentary systems in the platelet activation process, being (i) biochemical factors (adhesive molecules and extra-cellular matrix proteins, as well as soluble agonists) and (ii) biomechanical factors, such as hemodynamic shear forces (4, 5).

While the biochemical factors mediating clot formation have been extensively investigated, the vital role of the local fluid mechanical microenvironment that facilitates platelet-ligand interactions, thus regulating the release of biochemical cues, must not be overlooked in the development process of effective anti-thrombotic strategies. In particular, the mechano-sensitive plasma protein von Willebrand factor (VWF) and its binding with platelet receptor glycoprotein Ibα (GPIbα) play a crucial role in hemostasis, yet is also key to pathological thrombus initiation and propagation. Here, we discuss the mechanosensitive interaction of VWF with GPIbα, its dependency on shear forces generated by blood flow and possible therapeutic approaches exploiting this protein axis. Furthermore, novel shear-sensitive drug delivery platforms, designed to specifically target occluded vessels, are also discussed in this review.

## The Traditional, Biochemical Cascade of Thrombosis

Upon vascular injury, platelets adhere to exposed sub-endothelial matrix proteins, mainly VWF and collagen, followed by their activation, aggregation and finally formation of a “platelet plug” at the injury site. Under normal physiological conditions, the pro-thrombotic hemostatic response is neutralized by counter anti-thrombotic and anti-coagulatory mechanisms that confine the clot formation specifically to the injury site. However, at diseased vascular sites (such as atherosclerotic lesions) this balance is disrupted due to the highly thrombogenic contents and the stenotic geometry of the lesions, which cause an increase in local shear rates. The exacerbated biochemical and biomechanical responses combined can lead to abnormal thrombus propagation, turning a life-saving defense mechanism into a life threatening one.

In the traditional concept of platelet aggregation, soluble-agonists such as ADP and thromboxane A2 (TxA2) were assumed to play the central role in driving thrombus development. As per this model, the process of thrombogenesis and propagation comprised of three sequential steps, including platelets adhesion, activation, and subsequent aggregation at the site of vascular injury.

### Platelet Adhesion

The initial arrest of platelets at the site of vascular damage is primarily facilitated by shear-dependent interaction of platelet receptor GPIbα with the A1 domain of VWF (6, 7). The GPIbα-A1 binding is also key for platelet aggregation during thrombosis, which will be discussed in detail later. Other transmembrane platelet receptors mediating platelet adhesion include GPVI (8) and integrin α_2_β_1_ (9), that bind directly to collagen (10).

### Platelet Activation

The adhesion of platelets to sub-endothelial matrix proteins leads to platelet intracellular signaling, which activates the cell. Briefly, platelet activation results in conformational change (from the typical discoid shape to an elongated form) (11) and degranulation (i.e., release of the platelet cytoplasmic granules content) (12). α-granules release VWF and coagulation factors, such as fibrinogen, factor V, factor XIII, and P-selectin (also expressed on the platelet surface following activation); Dense granules release platelets agonists, such as serotonin and adenosine diphosphate (ADP) (13). This process results in sustained, oscillatory cytosolic Ca^2+^ flux, production of TxA_2_, exposure of phosphatidylserine (PS), expression of P-selectin, and microvesiculation, allowing coagulation factors to bind the activated platelet. The activation step also involves additional platelet recruitment via an outside-in signaling, induced by a paracrine action of the soluble agonists released from activated platelets themselves (e.g., ADP, TxA_2_) or by activated coagulation factors, such as thrombin. While the purinergic receptor P2Y_12_ is the platelet receptor for ADP, protease-activated receptors (PAR) 1 and 4 bind and potently react to thrombin (12, 14, 15).

### Platelet Aggregation

Following platelet adhesion and activation, the integrin α_IIb_β_3_ (also known as GPIIb/IIIa) undergoes conformational change through inside-out signaling and binds fibrinogen (16, 17). This receptor-ligand interaction results in the formation of platelet aggregates, ultimately forming a platelet plug that can prevent bleeding in case of minor injuries. However, for a damage of a greater magnitude, primary hemostasis is followed by secondary hemostasis, in which fibrin production stabilizes the clot to prevent hemorrhage (18). Since only initially-adhered platelets are in direct contact with thrombogenic surfaces, post-activation signals originating from platelets themselves as well as soluble agonists play a central role in triggering ongoing thrombus growth.

It has long been established that thrombus formation occurs via an interplay between two distinct, yet complementary platelet aggregation mechanisms: soluble agonist-dependent (biochemical) and rheology-dependent (biomechanical) pathways. However, major anti-thrombotic drugs target the biochemical axis of platelet aggregation without considering the biomechanical aspect. As a result, current anti-platelet drugs not only have limited clinical efficacy in treating cardiovascular diseases, but also carry significant side effects, that can be life-threatening at times. Thus, there is a pressing need to develop novel drugs that target growing thrombi specifically under pathological shear forces.

### Limitations of Current Anti-platelet Drugs

Anti-platelet drugs have proven to be the keystone of anti-thrombotic therapy, as demonstrated by multiple, large-scale clinical trials and meta-analyses (19, 20). Despite being a mainstay in preventing atypical platelet activation/aggregation in cardiovascular events, anti-platelet drugs face several limitations in the clinical settings. These include heterogeneity in interpatient response with considerable drug resistance (21–24), delayed onset of action (25, 26), low safety profile (27, 28) and systemic administration nature, resulting in limited effective concentration at the site of interest (29, 30). However, the most crucial side effect of antiplatelet drugs is a bleeding risk, that can have serious implications for the patient, including death (31–34). Conventional anti-platelet drugs blocking GPIIb/IIIa (abciximab, eptifibatide, tirofiban) or targeting TxA_2_ (dazoxiben, ifetroban, dipyridamole) and ADP-P2Y_12_ pathways (ticagrelor, cangrelor) have been associated with enhanced hemorrhagic episodes due to undesired impairment of hemostasis in addition to the desired prevention of arterial thrombosis (35, 36). Further, prolonged use of some cyclooxygenase-2 (COX-2) inhibitors, such as valdecoxib, parecoxib, and celecoxib, has been found to increase the recurrence of serious cardiovascular events in patients (37, 38).

### Dual Antiplatelet Therapy and Triple Therapy

The Clopidogrel in Unstable Angina to Prevent Recurrent Events (CURE) trial demonstrated the advantages of dual antiplatelet therapy (DAPT), including aspirin and P2Y_12_ receptor antagonist (clopidogrel, prasugrel, or ticagrelor) in acute coronary syndrome (ACS) patients over aspirin monotherapy (39, 40). Despite vast evidence supporting the usage of DAPT over aspirin alone in ACS or post-percutaneous coronary intervention (PCI), clinicians have not reached a consensus on the optimum duration of the therapy in these patients (41). Short-term DAPT is typically less effective in attenuating thrombotic events, but has lower bleeding risk. In contrast, long-term DAPT may reduce ischemic risk but increase the occurrence of major bleeding events, with a higher all-cause mortality rate (42, 43).

Triple therapy, including aspirin, P2Y_12_ antagonist and oral anticoagulant has shown a 3-fold increase in bleeding events, as compared to oral anticoagulant alone (44, 45). Generally, intensification of antithrombotic regimens for a maximal clinical benefit in a broader patient population comes at a major cost of significant bleeding events that may be proven fatal at times. Refining the anti-thrombotic effects of a drug without exacerbating the bleeding risks remains a major challenge to date.

In conclusion, current antiplatelet therapeutics are unable to meet the major clinical need to selectively prevent pathological thrombosis without interfering with the physiological process of hemostasis. As mentioned above, one of the significant distinctions between the two processes is the substantial difference in local shear rates, with exceedingly high shear levels observed in pathological thrombi at sites of vessel occlusion or atherosclerotic plaque rupture (46, 47). No existing, clinically-used antiplatelet drug is known to specifically respond to this biomechanical force at the site of pathological thrombus formation.

## Biomechanical Aspect of Thrombosis

### Shear Forces Resulting From Blood Rheology

Blood, circulating in the vessels via pressure differences, exerts forces on its components as well as the vascular walls (48). Blood flow is laminar, with maximal velocity at the vessel lumen center and zero at the vessel wall. The velocity of circulating blood changes differentially between its discrete fluid layers, thereby generating a tangential force (force acting along a tangent to the object) between them. This tangential force per unit area between laminae, termed “fluid shear stress” (ζ), is expressed in Pascal (Pa). Alternatively, wall shear rate (γ, expressed in s^−1^), defined as the rate of change in fluid velocity as a function of distance from the vessel wall, is used to describe hemostasis and thrombosis. In veins and larger arteries experiencing high shear rates, blood exhibits the characteristics of a Newtonian fluid, with the resulting wall shear stress directly proportional to the local shear rate (49).

The biomechanical forces generated by the virtue of blood flow play a vital role in uniting or separating the essential hematological components engaged in clot formation. Under normal hematocrit conditions (~40%) (50), erythrocytes mainly circulate along the central axis of the blood vessel due to axial migration. Consequently, platelets travel in close proximity to the vessel walls, a phenomenon that facilitates their binding to adhesive ligands at the reactive endothelial layer in damaged vascular sites (49, 51–53).

Under physiological conditions, typical wall shear rates of 300–800 s^−1^ occur in the large arteries, 15–200 s^−1^ in veins and 450–1,600 s^−1^ in micro arterioles (54). However, vessel stenosis due to atherosclerotic lesions or pre-existing thrombi can increase shear rates well beyond 10,000 s^−1^, resulting in a localized pro-thrombotic microenvironment (48, 54, 55).

### Platelet Adhesion and Tethering to VWF Under Shear Conditions

The arrest of flowing platelets occurs on immobilized VWF via interactions between the A1 domain of VWF and the platelet receptor GPIbα. These bonds have a rapid on-rate but short lifetimes, hence only supporting transient platelets adhesion and classical stop and go motion (56). Membrane tethers, consisting of smooth cylinders of lipid bilayer, then extend from the platelet surface and are pulled by shear forces. Membrane tethers are vital in prolonging platelet adhesion times, allowing for small platelet aggregates to form, which in turn maintain high local concentration of activating signals by eradicating any “washout effects” of soluble agonists by blood flow.

Tethers act as adhesion contacts between platelets and platelet-matrix, and subsequently facilitate bonding between the α_IIb_β_3_ platelet receptors and the Arg-Gly-Asp (RGD) motif of the VWF C1 domain (57). In normal physiology, under low-shear regimes (600–900 s^−1^), the aggregation is dominated by α_IIb_β_3_-dependent interactions, while under intermediate shear rates (1,000 to 10,000 s-1) both GPIbα and α_IIb_β_3_ play an important role in platelet aggregation. However, VWF-dependent interactions become increasingly dominating as shear rates increase (>10,000 s^−1^) (4, 58, 59). Consequently, VWF and GPIbα play a vital role in hemostasis but an even more pivotal role in the onset of thrombosis, thus contributing in several diseases.

### VWF Structure, Native Confirmation, and Pathophysiological Significance

Von Willebrand Factor is a large, blood-borne multimeric glycoprotein, crucial for hemostasis and thrombosis. It is secreted by both endothelial cells (where it is stored in Weibel-Palade bodies) and megakaryocytes (that also pack VWF in α-granules in platelets) as a globular multimer, consisting of identical subunits of 250 kDa, connected via disulfide bonds to form ultra-large VWF (ULVWF), weighing up to 20,000 kDa. Each subunit of ULVWF harbors multiple functional domains, each with a specific function. The major functionalities of plasma VWF are exhibited by three adjacent A domains, namely A1, A2, and A3. While A1 is primarily responsible for binding with the platelet receptor GPIbα, A3 acts as the immobilization site for VWF on the collagen matrix (60, 61). A2 is responsible for size regulation of ULVWF in blood physiology, as described below. Under shear forces, self-association of plasma VWF occurs also on the surface of platelets (62) and endothelial cells (63), thereby creating fibrillar structures that further facilitate platelet adhesion. As mentioned earlier, VWF is physiologically and pathologically important protein. It is responsible for several hemorrhagic disorders, including von Willebrand disease (VWD) and Bernard Soulier syndrome. VWD is the most common, hereditary bleeding disorder, resulting from qualitative or quantitative deficiencies in VWF, presiding a diverse range of mild to severe hemorrhagic episodes. VWD can be categorized into three types: VWD type 1, a quantitative defect; VWD type 2, a qualitative defect and VWD type 3, the most severe defect characterized by complete absence of VWF. VWD type 2 can be further divided into four sub-types, including type 2A (dominant loss-of-function mutations), type 2B (dominant gain-of-function mutations), 2M (impaired binding to GPIbα) and type 2N, or Normandy defect (impaired binding to factor VIII) (64). Moreover, acute coronary syndrome (ACS), acute myocardial infarction, stroke and ischemic events have been associated with increased levels of plasma VWF in several population-based studies (65, 66). Other studies have suggested that the single nucleotide polymorphisms (SNPs) on the gene coding for VWF may lead to coronary heart diseases in advanced atherosclerotic and diabetic patients (67, 68). Histopathological studies have also identified the pathogenic role of VWF in venous thromboembolism (VTE) by revealing VWF-rich thrombi in patients who died from the disease (69).

### Shear-Induced Activation and Conformational Change of VWF

Following secretion from Weibel-Palade bodies in the endothelium, ULVWF multimers (several 100 μm long) are inherently hyperactive as they carry numerous ligand binding sites and are more receptive to hemodynamic forces due to their size (70).

**Figure 1:**
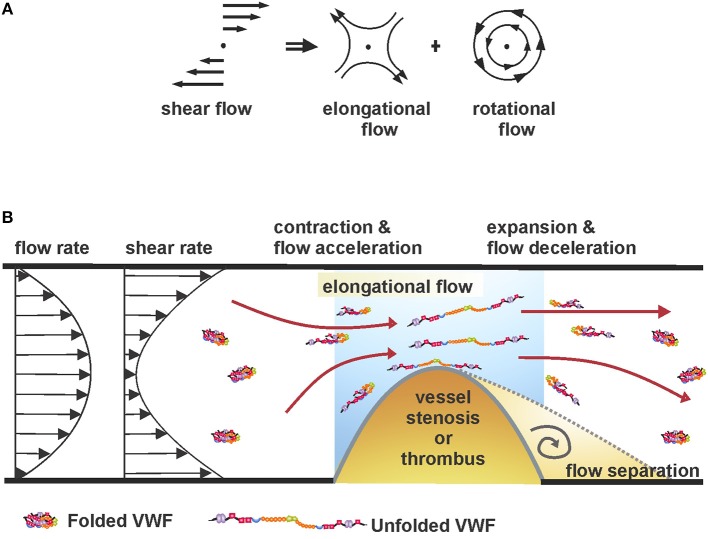
**(A)** Shear flow, as a superimposition of rotational and elongational flow components (71, 72). **(B)** Unfolding of coiled VWF under elongational flow or shear rate gradients at sites of vessel stenosis.

The shear force is comprised of rotational and elongational flow components (Figure 1A) (71, 72). Under low shear forces, VWF exists in “coiled” or globular conformation due to monomeric self-association (73). In this native conformation, binding sites in the A1 domain are believed to be buried and thus, inaccessible to platelets or other biomolecules (74, 75). Under flow-induced shear stress, VWF exhibits conformational variation, including end-over-end tumbling and periodic elongation and compaction (76, 77). Indeed, above a critical shear rate, the intrinsic interactions between the monomeric units are overcome by the hydrodynamic drag, resulting in stretching of VWF, thereby exposing the cryptic A1 epitopes (77, 78). While the critical shear rate to elongate surface-immobilized VWF is about 3,000 s^−1^, circulating plasma VWF requires ~5,000 s^−1^ (79, 80). Interestingly, the required force for VWF elongation increases with increasing shear rates (77).

At sites of vessel constriction, fluid acceleration and subsequent deceleration create local force gradients. As a result, VWF circulating nearby experiences an elongational pull in the direction of flow due to a faster-moving proximal end and a slower-moving distal end (Figure 1B). Importantly, studies have shown that the unfolding process of VWF can be achieved at significantly reduced flow rates if they produce a gradient of shear forces (i.e., when flow is accelerating or decelerating), compared to a flow with high, but constant shear rate (81, 82). The is because of the large size of VWF where the leading end of the molecule will experience a different strain rate compared to the trailing end, hence substantially lower forces are required to unfold the protein (76).

### Shear-Sensitive VWF A2 Domain and Mechanoenzymatic Cleavage

ULVWF concatemers (long molecules containing multiple copies of the same protein sequence, linked end-to-end in series; Figure 3) remain tethered to the endothelial cell surface in stretched conformation with exposed A2 domain that completely unfolds due to missing long-range disulfide linkages, unlike A1 and A3 domains (84). On complete refolding of the A2 domain, a peptide bond, Tyr1605 -Met1606, located in the centrally situated β4 sequence in the β-sheet (at the center of the folded A2 domain), is recognized by ADAMTS-13, a metalloprotease enzyme (81). ADAMTS-13 cleaves ULVWF at this scissile bond and releases the smaller and less hemostatically active VWF strings into the blood stream. This regulatory mechanism prevents VWF hyperactivation under physiological shear rates. Severe deficiency of ADAMTS-13 activity caused by inhibiting autoantibodies results in ultra-large multimers of VWF in blood. These ULVWF cause systemic microvascular thrombosis, ultimately leading to a blood disorder called thrombotic thrombocytopenic purpura (TTP) (85). Single molecule studies found that proteolysis of VWF is regulated by a Ca^2+^ binding α3-β4 loop in the A2 domain. Therefore, within the physiological calcium concentration range the refolding kinetics of A2 can increase five times without affecting the unfolding event (86).

### Shear-Sensitive VWF A1 Domain and Platelet Arrest

The VWF A1 domain plays a remarkable role in initiating thrombus formation under pathological shear rates. Under low shear conditions or absence of any exogenous VWF modulators, such as ristocetin and botrocetin, no interaction occurs between VWF and platelet surface receptors. However, above threshold shear levels (800 s^−1^ in humans), the exposed A1 domain of unfolded VWF—immobilized onto exposed collagen—binds GPIbα receptors in (GP)Ib-IX complex on platelets cruising within the “latching distance” of the vascular wall, thus initiating platelet adhesion to VWF and aggregate formation (57, 59, 87–89).

A1 domain is the most positively charged domain of VWF due to the presence of Cys509-Cys695 disulfide bond, a major positively charged region surrounded by two discontinuous anion flanking sequences, which contains seven sialylated glycosylation sites (74). Studies using artificial peptides indicated that residues Asp514-Glu542 within this disulphide loop in the A1 domain could be involved in GPIbα binding (90). Ristocetin, an antibiotic from *Nocardia lurida*, activates VWF and facilitates A1-GPIbα interaction in a similar fashion as elevated shear. Ristocetin binds to proline-rich sequence, Glu700 to Asp709 and the C-terminal to the Cys509-Cys695 disulphide bond in the A1 domain. In some studies, anti-VWF A1 (5D2, CR1, and 6G1) and anti-GPIbα monoclonal antibodies (AK2 and AN51), mapping ristocetin-bound A1 and GPIbα epitopes, selectively inhibited both ristocetin-induced and shear-induced platelet aggregation (74, 91).

Finally, the A1 domain also acts as the main binding site to non-fibrillar collagen type VI (92). Like the A3 domain, which serves as the main binding site for collagens I and III, the A1 domain can bind both types, although with less propensity. In fact, under high shear forces, the unraveled A1 domain can act as surrogate for the A3 domain in recruiting platelets to collagen.

### Force-Induced A1 Activation

**Figure 2:**
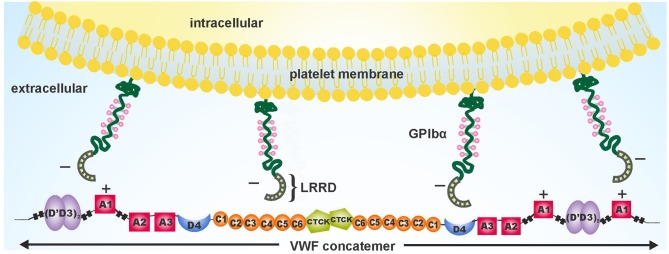
Schematic depiction of the electrostatic complementary interactions between negatively charged platelet surface receptors GPIbα and positively charged A1 domains exposed on fully unfolded VWF. Adapted and modified from Fu et al. (83).

Recent study described a two-step conformational transformation mechanism of A1 activation after VWF senses mechanical force (83). The first step involves elongation of VWF, immobilized to the vessel wall, from a globular form to a stretched form, followed by a tension-dependent local transition to a high affinity state. Elongation results in breaking of weaker hydrogen bonds between distal monomers in the tethered VWF concatemer. The molecule then experiences a tensile force upstream, along the backbone toward the tether point. This exposes an almost linear array of A1 domains that become easily accessible to platelets. When this tensile strength reaches ~21 piconewton (pN) in the upstream regions, A1 transforms into a second state exhibiting high affinity for GPIbα, that depends upon electrostatic interactions [Figure 2; (83)].

### Platelet GPIbα and A1 Binding

At the onset of hemostasis and thrombosis, platelets tether or translocate onto the VWF substrate immobilized on the damaged vessel site. The initial contact between circulating platelets and VWF is made via binding interactions between the A1 domain of VWF and the N terminal domain of GPIbα receptor (56, 57). In physiology, the levels of ligand-receptor interactions are regulated, as insufficient interactions may affect hemostasis by causing bleeding while excessive interactions may lead to thrombosis.

### GPIbα Receptor

GPIb-IX-V receptor complex is abundantly expressed with about 25,000 copies of GPIb-IX complex and 12,000 GPV copies on resting platelets, making it suitable for platelet adhesion and aggregation (93). The complex consists of GPIbα, GPIbβ, GPIX, and GPV sub-units in the stoichiometric ratio of 2:4:2:1 (94). GPIb complex comprises of GPIbα and GPIbβ, connected via disulfide bond that tightly link with GPIX to form GPIb-IX complex. GPV does not participate in VWF binding or signaling (95). GPIbα consists of a N-terminal leucine-rich repeat domain, a macro glycopeptide region followed by a long stalk, a C-terminal transmembrane helix and a short cytoplasmic domain (96). Polymorphisms in GPIbα have been linked with a greater risk of cardiovascular manifestations, such as stroke and myocardial infarction in young individuals (97, 98).

### GPIbα-VWF A1 Binding Kinetics

**Figure 3:**
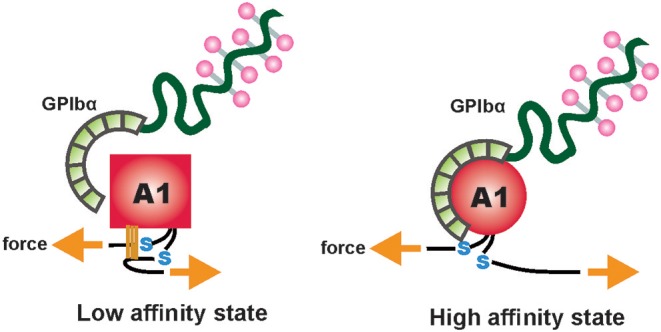
A two-state model depicting flow-induced activation of the A1 domain of VWF, tethered to the vessel wall. Hydrogen bonds between residues, internal and external to the A1 disulfide link are disrupted due to mechanical tension under shear flow. This results in conversion of the A1 domain from a low-affinity, flexed state to a high-affinity extended state. Adapted and modified from Fu et al. (83).

Crystal structures of the VWF A1-GPIbα complex, both of wild-type or a gain-of-function A1 and GPIbα mutations, exhibited two distinct binding interfaces (99). The leucine rich repeat domain (LRRD) of GPIbα [basal isoelectric point (pI): 5.87] is a negatively charged, concave- or horseshoe-shaped region, that binds with the positively charged VWF A1 domain (basal pI: 9.4) via electrostatic interactions [Figure 3; (99)]. The electrostatic complementarity of the binding interfaces enhances the association rate of A1-GPIbα complex (100).

Shear flow modulates the dissociation rates of the A1-GPIbα bond. Earlier studies hypothesized that at forces <22 pN, A1-GPIbα exhibits a “catch” bond behavior, in which bond lifetime prolongs as shear forces increase (translating as tensile forces). Above 22 pN, the bond acts as a “slip” bond, where increasing shear results in shortened bond lifetime (101, 102).

Later studies using Receptor and Ligand In a Single Molecule (ReaLISM) constructs described the A1-GPIbα bond as a mechanically stabilized “flex bond.” The bond exists in two “slip” bond states, one flexed, low-affinity state associated with lower force and the other extended, high-affinity state engaging ~10 pN with around 20-fold longer bond lifetime and greater ability to withstand strong hemodynamic forces (103). Tensile force stabilizes the high-affinity state, thus strengthening the bond under pathological shear rates. Similar phenomenon was observed for ristocetin, a VWF modulator that alters the conformational state of the A1-GPIbα bond in a sheared-like configuration. Both ristocetin and tensile force selectively stabilize the extended state, corresponding to shear-induced state (74, 91, 103). Formation of mechanically stabilized flex-bonds may account for the resistance of A1-GPIbα complex under extreme pathological shear rates, resulting in platelet adhesion and activation-independent platelet aggregation at these extreme shear conditions (80). Interestingly, the VWF A1 domain with type 2B mutations forms “slip” bonds with platelet GPIbα, resulting in prolonged bond duration even at low shear rates (101).

### GPIbα Mechanosensing and Mechanotransduction

Recent findings employing biomechanical techniques at the single molecule level have provided insight into how platelets mechanosense shear forces and subsequently convert these mechanical cues into biochemical signals to promote thrombus growth and stability. A structured, quasi-stable Mechano-Sensitive Domain (MSD), consisting of around 60 residues, was identified in the juxtamembrane stalk region of GPIbα (104). VWF A1-mediated mechanical pulling on the GPIbα N-terminal domain results in unfolding of the MSD in GPIb-IX complex. Furthermore, this shear-mediated pulling of tethered platelet onto immobilized VWF generates tensile force along GPIbα that not only unfolds the relatively unstable MSD, but also lowers the overall force applied on A1-GPIbα complex, in turn stabilizing the tethering of platelet. MSD unfolding occurs at similar force range (5–20 pN) as that required for the A1-GPIbα complex to transition into high-affinity flex-bond state (101, 103). Due to direct contact of the MSD with GPIbβ and GPIX subunits, unfolding of the mechanosensitive domain may induce conformational changes in these subunits, thereby facilitating intracellular signal transmission (104).

In mutational studies with human/canine chimeras of GPIbα, co-crystal structure of the A1-GPIbα complex showed minimal contact between the predominantly negatively charged LRR2-4 (Leu60-Glu128) sequence and VWF A1 under static conditions. Interestingly however, this sequence was not only important for ristocetin-induced binding but also increasingly fundamental for platelet adhesion at increasing shear rates. This finding further supports the idea of force-mediated transition from low to a stabilized, high-affinity state of ligand-receptor flex-bond wherein, the LRR2-4 of GPIbα establishes tight contact with the A1 domain (105, 106). Yet, the conformational dynamics of the LRRD and its role in signal transduction within platelets is not well-understood.

Recently, using molecular dynamic simulations and biomembrane force probe, unfolding of LRRs in the N-terminal domain of GPIbα was observed under force application, initiating at LRR2-4 and propagating to neighboring LRR sequences. LRR folding prolongs the A1-GPIbα bond lifetime by increasing the force required for its disconnection from 10 to 25 pN (i.e., a “catch” bond behavior) and reduces the bond lifetime thereafter (a “slip” bond behavior) as described earlier in section GPIbα-VWF A1 Binding Kinetics (101, 107).

Thus, far, the GPIbα mechanosensing was recapitulated by the conformational dynamics of MSD and LRRD. However, mechano-transduction or how the mechanical cues were transformed into biochemical signals remained elusive. Ju et. al. demonstrated that mechanotransduction of force into signals was done by cooperative interplay between the mechanosensing domains MSD and LRRD (108). The study showed that unfolding of LRRD enhances the unfolding of MSD by force transmission through mucin-like macroglycopeptide located between the two domains. MSD unfolding event may aid further unfolding of LRRD, thus prolonging the A1-GPIbα bond lifetime. This cooperativity pattern between the mechanoreceptor domains is maximal around the optimal force of 25 pN [108].

Single platelet calcium imaging identified three types of Ca^2+^ fluxes: Null-type, baseline with background noise; α-type, latent phase following a high spike with a rapid decay; and β-type, being fluctuating or slowly increasing Ca^2+^ signals to an intermediate level followed by slow decay to the baseline. GPIbα-A1 binding results in transient α-type Ca^2+^ flux, promoting reversible arrest of translocating platelets on immobilized VWF (92, 109). Time-lapse correction analysis revealed that while LRRD unfolding enhances the Ca^2+^ flux, MSD unfolding event was essential to generate α-type Ca^2+^ signal. In resting platelets, a signaling molecule−14-3-3ζ–is bound to both GPIbα and GPIbβ (110). Upon A1-GPIbα binding, the head domain of GPIbβ binds with the unfolded MSD while detaching its cytoplasmic tail from GPIbα-associated 14-3-3ζ. 14-3-3ζ is proposed to transduce the mechanical signal of MSD unfolding into intracellular biochemical signaling across the platelet membrane. This leads to the downstream activation of adapter molecules, ultimately resulting in cytoplasmic calcium flux [108]. In addition, GPIbα-A1 interactions lead to other intracellular signaling events, including integrin α_IIb_β_3_ activation, ADP, thrombin and TxA2 production and P13K activation.

### Integrin α_IIb_β_3_ (GPIIb/IIIa) Activation

GPIbα mechano-signaling results in a transient calcium flux that thrusts the majority of integrins to an intermediate activation state. This intermediate state subsequently facilitates the outside-in signaling of α_IIb_β_3_ integrins for further transformation into an active state. These intermediate and active integrin states have longer bond lifetimes and higher ligand affinity that stabilize the platelet aggregates (111). The high-affinity state of integrin α_IIb_β_3_ enables its binding with fibrinogen and VWF to strengthen the adhesive bonds among platelets and stabilize aggregation.

### Shear Dependent Platelet Aggregation: Evolving Concepts From Constant Shear to Gradients of Shear

Distinct aggregation mechanisms operate based on different shear ranges (5, 112). α_IIb_β_3_-fibrinogen-dominated platelet aggregation occurs mainly under low shear rates (<1,000 s^−1^) in veins and larger arteries, with soluble agonists maintaining the activated state of integrins, thus stabilizing the bonds. Platelet-platelet interactions become increasingly dependent on VWF and its binding with both α_IIb_β_3_ and GPIbα receptors at increasing shear range (1,000–10,000 s^−1^), as observed in moderate arterial stenosis and arterioles. Notably, at pathological shear rates (>10,000 s^−1^), resulting from acute vessel constriction, initial aggregation exclusively relies upon VWF-GPIbα bonding, without the prerequisite of platelet activation or α_IIb_β_3_ interaction (5, 80). Above this shear threshold, soluble VWF binds with GPIbα receptors on adherent platelets, resulting in dominating platelet-platelet interactions that induce “large rolling aggregates” onto immobilized VWF. Enhanced inter-platelet interactions mediated by soluble VWF result in a growing transient aggregate under extreme shear conditions (80). A dramatic increase in shear may also destabilize vulnerable thrombi formed post-plaque rupture, generating emboli that can flow to distal organs, causing partial or complete vessel blockage downstream of plaque origin (113).

The mechanisms of platelet aggregation described up until this point operate under simplified laminar shear conditions, with constant shear and no turbulence in consideration. However, in pathology, blood rheology can alter dramatically, both spatially and temporally, in the local microenvironments generated by vascular lesions, acute stenosis or pre-existing thrombi. Thrombus propagation within the blood vessel is a complex and dynamic process that can incur flow perturbations, resulting in vortex and eddy formation, shear gradients, recirculation zones, flow separation and reciprocation (114, 115). These disturbed flow parameters have substantially stronger prothrombotic effects as compared to constant shear flows of the same magnitude.

### Platelet Aggregation Mechanisms Under Complex Shear Gradient: Novel Concepts

Elegant studies both *in vitro* and *in vivo* have emphasized the indispensable role of shear gradient-dependent functionality of VWF in recruiting platelets for thrombus propagation. As mentioned above, thrombus development under high shear conditions is an intricate process that is principally driven by hemodynamic forces. Vessel stenosis can create a microenvironment wherein the forces experienced by blood components vary vastly as compared to any other non-obstructed sections of the vasculature.

**Figure 4:**
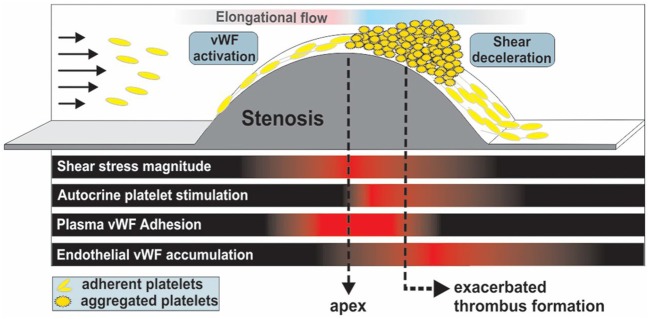
Thrombus formation and propagation is aggravated post-stenosis (simulating atherosclerotic-like geometries) in a VWF-dependent fashion due to shear deceleration, autocrine platelet stimulation and elevated VWF deposition at the stenosis outlet. Adapted with permission from Westein et al. (120).

Pathological shear rates briefly exceeding 10,000 s^−1^ can be generated locally by occlusive thrombi at the sites of atherosclerotic plaque ruptures, causing an exaggerated aggregation response by platelets, thus precipitating myocardial infraction or ischemic stroke (24, 116). A landmark study by Nesbitt et al. (117) elucidated the principle role of shear microgradients, resulting from vascular constriction, in promoting stabilized, discoid platelet aggregation and thrombus progression post-stenosis. Shear gradient regions consist of three zones: a shear acceleration zone at the proximal end of stenosis, a peak shear zone at the apex of stenosis and a shear deceleration zone at the distal end of stenosis. While the high shear region enhances platelet activation (118, 119), the low shear region post stenosis provides sufficient time and conducive flow conditions for platelet hyper-aggregation and thrombus progression [Figure 4; (121–123)]. Computational Fluid Dynamics (CFD) modeling of stenosed vessels showed that for an input shear rate of 1,800 s^−1^ upstream of the stenosis, a shear rate 20,000 s^−1^ is generated at the apex with rapid deceleration to about 800 s^−1^ post-stenosis, inducing exacerbated platelet aggregation. Interestingly, no thrombus has developed under similar conditions with a constant high shear rate of 20,000 s^−1^ (117). As it turns out, platelets experiencing sudden and steep shear acceleration form transient aggregates without prior activation or shape change, by tethering onto immobilized VWF with the boost of soluble VWF. Labile interactions between VWF and GPIbα result in rapid translocation of platelets into downstream region with much lower shear rates. Marked reduction in shear forces in the deceleration zone results in α_IIb_β_3_ engagement corresponding to localized Ca^2+^ spikes. Ca^2+^-dependent signaling causes restructuring of filamentous platelet tethers into bulbous morphology, which ultimately stabilizes discoid platelet aggregates. On the thrombus surface, platelets remain loosely adhere via GPIbα without any activation. On the contrary, platelets consolidate in the stable thrombus core via α_IIb_β_3_ interactions following weak activation (117).

Westein et al. further elucidated the process of shear gradient-dependent thrombus formation in atherosclerosis by characterizing the central role of VWF in promoting platelet aggregation post-stenosis in both *in vivo* stenosis models of damaged murine carotid arteries and *in vitro* microfluidic perfusion assays emulating arterial flow (120). Shear micro-gradients or elongational flow unfold VWF at forces two orders of magnitude lower compared to constant shear, enhancing platelet recruitment substantially even at low venous flow rates (82). Adhesion of a few circulating platelets by elongated VWF immobilized at the stenosis apex can create a snowball effect with hyper-aggregation of platelets, leading to full vessel occlusion in minutes. Significantly, at shear stress exceeding 100 dynes/cm^2^, wherein VWF takes charge of platelet recruitment, aspirin, the gold standard of antiplatelet therapy, is not able to inhibit platelet aggregation, as shown in multiple studies (124–126). Selective COX-2 inhibition even enhanced platelet aggregation under elevated arterial shear stress by reducing the basal production of prostacyclin (prostaglandin I2; PGI_2_), a potent inhibitor of platelet aggregation especially at high shear stress (127, 128).

Overall, it is clear that the shear environment at sites of thrombus formation has a profound effect on various aspects of platelet behavior. In particular, high shear forces induced by the architecture of large thrombi create a feed-forward mechanism that can precipitate occlusive thrombus formation. As such, a better understanding of the shear-dependent aspects of thrombus formation will aid in the design and development of future anti-thrombotic therapies.

## Therapeutics Targeting Shear-Dependent Thrombus Formation

As discussed in the previous sections, shear-dependent VWF unfolding is crucial for initial platelet adhesion, followed by their aggregation and potential formation of a pathological thrombus. Failure of current antithrombotics to effectively prevent pathological thrombosis without bleeding side-effects has been a major obstacle in combating cardiovascular diseases.

Therefore, targeting pathological shear rates that are uniquely present at sites of thrombus development using “smart” therapeutics can be highly effective in preventing total vessel occlusion, significantly improving the efficacy and safety profile of anti-thrombotic therapy.

Two approaches of preventing occlusive thrombi by exploiting pathological shear conditions are discussed hereafter. One approach would be to inhibit shear-driven interaction of the VWF A1 domain and GPIbα. The second approach involves shear-sensitive vehicles that specifically target occlusive thrombi due to the abnormally-high shear rates surrounding this environment.

### Therapeutics Targeting Shear-Driven VWF A1-GPIbα Interaction

**Figure 5:**
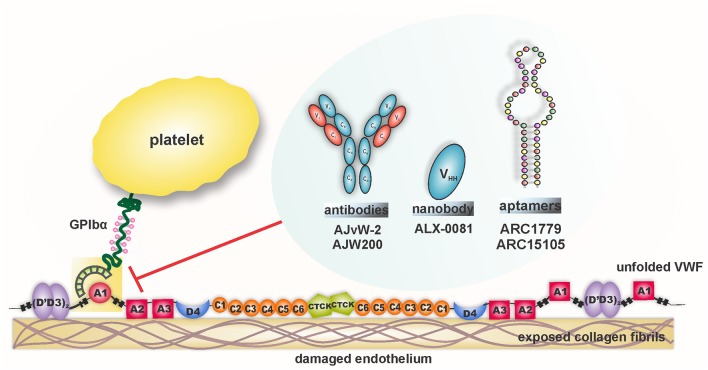
Therapeutics targeting and inhibition of shear-driven VWF A1-GPIb interaction.

Considering the diverse pathophysiological impact of the VWF-GPIbα interaction, this ligand-receptor pair has been an attractive target in the evolutionary field of antithrombotic therapy. Undeniably, VWF's foremost interaction with the circulating platelet is shear-mediated; therefore, blocking this interaction at pathological shear rates is anticipated to inhibit hyper-aggregation of platelets in the diseased blood vessel, leaving the healthy vessels unaffected. Assorted agents have been designed and tested to target VWF-GPIbα binding under shear conditions, as depicted in Figure 5. The results from preclinical and clinical studies are outlined in Table 1.

**Table 1:** Outline of approaches that inhibit VWF-GPIbα interaction specifically under high shear rates.

**Name**	**Molecule type**	**Injury model/organism/administration**	**Experimental outcome**	**Development phase**	**References**
AJvW-2	Murine monoclonal antibody	– Photochemically –induced thrombosis/guinea pigs/intravenous ACS patients with UAP/AMI Balloon injury/guinea pigs/intravenous	Inhibition of Ristocetin and Botrocetin-induced aggregation of human and guinea pig platelets; dose dependent inhibition of H-SIPA in human and guinea pig platelets, L-SIPA unaffected Thrombus prevention without prolonged bleeding times Inhibition of H-SIPA of acute ACS patients Dose dependent inhibition of thrombus and neointima formation	*In-vitro* *In-vivo* *In-vitro* *Ex-vivo*	(129) (129) (130) (131)
AJW200	Humanized monoclonal antibody	– cynomolgus monkeys/intravenous Balloon injury/Japanese white rabbits/intravenous Folts injury model/Beagle dogs/intravenous Humans (healthy)/intravenous	Inhibition of H-SIPA, adhesion, thrombin generation in human platelets Sustained inhibition of RIPA without extensive prolongation of the bleeding time in cynomolgus monkeys Inhibition of botrocetin-induced aggregation after first injury; reduction of cell proliferation thrombus and neointimal growth following second injury Thrombus inhibition in the stenosed coronary arteries without prolonged bleeding times Dose-dependent inhibition of RiCof activity, prolongation of PFA-100 closure time, no prolonging bleeding times, no significant adverse effects, no evidence of immunogenicity	*In-vitro* *Ex-vivo* *Ex-vivo* *Ex-vivo* Phase I	(132) (132) (133) (133) (134)
ARC1779	Aptamer	– Denuded porcine aorta segments Electrical injury arterial thrombosis model/cynomolgus monkeys/intravenous Humans (healthy)/intravenous Humans (patients with congenital TTP)/intravenous Humans (Patients undergoing CEA)/intravenous	Inhibition of botrocetin and high shear-induced aggregation in human platelets Reduction of platelet adhesion on collagen coated plates and thrombi formation in injured porcine arteries Dose-dependent inhibition of occlusive thrombus formation in cynomolgus monkeys with mild bleeding time prolongation Dose and concentration dependent VWF and platelet activity inhibition without major bleeding events or side effects Dose-dependent inhibition of VWF-related platelet function, stabilized platelet count in congenital TTP patients, Test doses insufficient to suppress all the clinical/laboratory features of TTP Reduction in cerebral embolic events in patients post CEA, Increased anemic and bleeding events	*In-vitro* *Ex-vivo* *In-vivo* Phase I Phase I/II Phase II	(135) (135) (135) (136) (137) (138)
ARC15105	Aptamer	Denuded porcine aorta segments cynomolgus monkeys/intravenous, subcutaneous	Better aggregate inhibition than ARC1779 under arterial shear conditions in perfusion chamber, >90% platelet activity reduction in segmented porcine arteries >90% VWF activity reduction in cynomolgus monkeys	*In-vitro/ex-vivo* *In-vivo*	(139) (139)
ALX-0081/ALX-0681 (INN: Caplacizumab/Cablivi)	Bivalent nanobody	– – Modified Folts injury model/baboons/intravenous Humans (Patients undergoing PCI)/intravenous mAb-3H9 (ADAMTS-13 inhibiting Ab) induced aTTP/Baboons/subcutaneous Humans (healthy)/intravenous Humans (stable angina patients undergoing PCI)/intravenous	Selective inhibition of platelet adhesion to collagen at high shear rates Complete inhibition of platelet adhesion on collagen in blood from PCI patients on standard antithrombotics Lower effective dose and bleeding risk, superior therapeutic window in baboons as compared to commercial antithrombotics Dose-dependent inhibition of platelet adhesion and aggregation in CAD patients prior to PCI, prolongation of col/ADP time in PFA-100 Complete neutralization of VWF activity without severe bleeding risk in thrombocytopenia Total RIPA inhibition at high dose in healthy subjects, no bleeding risk and immunogenic response reported, drug well-tolerated and safe Transient but clinically insignificant decrease in FVIII and VWF levels in patients, no increased bleeding risk or immunogenic response on dose increase even in addition to standard therapy, rapid clearance of unbound antibody	*In-vitro* *Ex-vivo* *In-vivo* *In-vitro* *In-vivo/ex-vivo* Phase I Phase Ib	(140) (140) (140) (141) (142) (140) (140)
		Humans (aTTP patients)/intravenous, subcutaneous Humans (aTTP patients)/intravenous, subcutaneous	More rapid resolution of TTP episodes, faster platelet-count normalization, higher frequency of complete remission and increased bleeding tendency as compared to placebo Faster resolution of TTP episode with shorter time to platelet count response, clinically significant reduction in aTTP recurrence, aTTP related deaths and major thromboembolic events as compared to placebo; higher bleeding-related TEAEs than placebo (Ongoing study)	Phase II (TITAN, NCT01151423) Phase III (HERCULES, NCT02553317) Phase IIIb (Post-Hercules, NCT02878603)	(143) (144) –

*CEA, carotid endarterectomy; PCI, Percutaneous Coronary Intervention; TTP, Thrombotic thrombocytopenic purpura; aTTP, Acquired Thrombotic thrombocytopenic purpura; H-SIPA, High shear-induced platelet aggregation; L-SIPA, Low shear-induced platelet aggregation; Ab, antibody; CAD, Coronary artery disease; RIPA, Ristocetin-induced platelet aggregation; RiCof, Ristocetin cofactor; ACS, Acute coronary syndrome; TEAEs, Treatment-emergent adverse events; UAP, Unstable angina; AMI, Acute myocardial infarction*.

#### AJvW-2

AJvW-2 is a murine, monoclonal IgG_1_ directed against the A1 domain of human VWF. It inhibits botrocetin- (IC_50_ 1.8 ± 0.3 μg ml^−1^), ristocetin- (IC_50_ 0.7 ± 0.1 μg ml^−1^), and high shear stress [10.8 Newton meter (Nm)^−2^]-induced aggregation, without affecting low shear stress (1.2 Nm^−2^)-induced aggregation of human platelets *in vitro*. The antibody prevented thrombus formation *in vivo* in photochemically-induced arterial thrombosis in carotid artery of guinea pigs, without prolonging the bleeding time (129). The efficacy of AJvW-2 on shear-specific inhibition of VWF-GPIbα interaction was tested in ACS patients (12 with unstable angina, 20 with acute myocardial infarction) with enhanced Shear-Induced Platelet Aggregation (SIPA). Using cone and plate viscometer, SIPA was measured for the patients' blood and the antibody was found to inhibit at 10 μg ml^−1^ high SIPA, without affecting aggregation at low shear stress (130). AJvW-2's prevention of thrombus generation and platelet adhesion within the carotid artery was also observed in guinea pigs, following a balloon catheter injury. Platelet aggregation was significantly inhibited for 2 days and neointima formation was circumvented 14 days following injury (131). The shear-specific blockade of VWF-GPIbα interaction by this monoclonal antibody provided it with an advantage of lower bleeding risk over GPIIb/IIIa antagonists.

#### AJW200

AJW200 is the humanized form of the anti-VWF monoclonal antibody, AJvW-2 (above), synthesized to overcome the immunogenicity and rapid clearance of AJvW-2 in humans. *In vitro* studies using cone and plate viscometer showed the selective suppression of thrombin generation, adhesion and aggregation of human platelets under high shear conditions, but not under low shear conditions. Moreover, the pharmacokinetic (PK) and pharmacodynamic (PD) profiles of AJW200 were investigated *ex vivo* in cynomolgus monkeys after intravenous administration. Dose-dependent inhibition of ristocetin-induced platelet aggregation was observed without extensively prolonged bleeding times as compared to abciximab (132). In the Folt's model of coronary arterial thrombosis in beagle dogs, significant inhibition of botrocetin-induced platelet aggregation and cyclic flow reductions were observed, but with marked bleeding time prolongation (133). Clinically, dose-dependent inhibition of platelet ristocetin cofactor (RiCof) activity was observed after AJW200 infusion along with an increased Platelet Function Assay (PFA)-100 closure time, with skin bleeding time augmentation (134). While the initial results with AJW200 seemed encouraging, data from the latter has not been published and no further clinical investigations with AJW200 have been reported.

#### ARC1779

ARC1779 is a synthetically manufactured aptamer (a short, single-stranded oligonucleotides) against the A1 domain of VWF, that strongly binds with its target (~2 nM dissociation constant) to prevent VWF-mediated platelet aggregation.

ARC1779 inhibited botrocetin-induced (IC_90_~300 nM) and high shear-induced (IC_95_~400 nM) aggregation of human platelets *in vitro*. In a study using electrically-induced arterial thrombosis in cynomolgus monkeys, ARC1779 inhibited occlusive thrombus formation with mild bleeding time extension (135). Overall, ARC1779 exhibited comparable antithrombotic efficacy as abciximab with lesser bleeding time prolongations. In a prospective, partial cross-over phase I/II clinical trial, safety and efficacy of ARC1779 were investigated in patients with congenital TTP (137). The results suggested that ARC1779 dose-dependently inhibited VWF-triggered platelet plug formation and stabilized the platelet count in TTP patients during infusion, with no associated bleeding events. However, the subcutaneous delivery of the aptamer yielded plasma concentrations that were insufficient to correct all the clinical or laboratory features of TTP (137). Later, a randomized, double-blind, placebo-controlled clinical study was performed in 36 patients undergoing carotid endarterectomy (CEA) to evaluate the immediate effect of ARC1779 on cerebral emboli post CEA (138). Embolic signals (ES), detected by transcranial Doppler ultrasound (TCD), were used to determine the effect of the drug. Rapid reduction of ES signals in terms of mean signal and frequency was seen by ARC1779. However, bleeding and anemic episodes were also reported in this study (138).

#### ARC15105

ARC15015 is the second generation, chemically modified, anti-VWF aptamer that was designed to target shear-dependent platelet aggregation in the clinical setting of myocardial infarction. Pegylated ARC15015 was developed to substantially enhance the suppression capacity of VWF activity, with increased bioavailability and half-life. ARC15105 completely inhibited ristocetin-induced platelet aggregation in whole blood and caused over 90% inhibition of platelet adhesion in denuded porcine aortic segments under high shear conditions. In PK and PD analysis in cynomolgus monkeys, the aptamer inhibited more than 90% of VWF activity without any spontaneous bleeds throughout the study (139).

#### ALX-0081/0681

ALX-0081 or Caplacizumab (INN) is the only drug candidate targeting the VWF A1 domain that has been approved by the FDA to treat adult aTTP patients (136). ALX-0081 is the first in class, humanized, bivalent nanobody that specifically binds to the GPIbα binding site on VWF. Nanobodies are derived from the heavy-chain variable domains (VHH) of naturally occurring camelid heavy chain antibodies. The bi-valency of ALX-0081 equips the nanobody with greater avidity to VWF. *In vitro* perfusion chamber studies, using blood from healthy individuals and PCI patients receiving standard-of-care treatment (aspirin, clopidogrel, and unfractionated heparin), showed the selective inhibition of platelet adhesion on collagen type III by the nanobody under high, arterial shear conditions (shear rates > 1,500 s^−1^), without any effect under low shear conditions. Plasma levels of 0.3 to 0.5 μg/ml were required for total inhibition of cyclic flow reductions (CFRs) by ALX-0081, corresponding to an effective concentration of 0.8 μg/ml in ACS patients and 0.4 μg/ml in healthy individuals in *ex vivo* experiments (145). The therapeutic window (the difference between the dose required for CFR and the dose resulting in enhanced bleeding rates) of ALX-0081 was found to be superior over abciximab and clopidogrel in baboons. Ristocetin-induced aggregation was inhibited by ALX-0081 in healthy individuals (at 0.4 μg/ml) and in Coronary Artery Disease (CAD) patients (at 0.8 μg/ml) with higher plasma VWF antigen levels. The nanobody also suppressed platelet adhesion to collagen (Col) under high shear rates and prolonged Col/ADP closure time in PFA-100, both in a dose-dependent fashion (141).

In the setting of TTP, the anti-VWF nanobody rapidly reversed the marked decline in platelet count, along with normalization of lactate dehydrogenase (LDH) levels (which correlates with VWF activity). Most importantly, no drug-related excessive bleeding risks were observed, regardless of reduced VWF activity and platelet count (142). In an open-label extension study, comprising stage C heart failure patients, an i.v. bolus injection of the drug at 6 mg was administered in 22 Stable Angina (SA) patients. Similar bleeding episodes were observed in the drug-treated and placebo-treated groups, thus proving that ALX-0081 did not result in a greater bleeding risk in these subjects, even in combination with standard antithrombotic therapy. Stable inhibition of VWF-mediated platelet aggregation was found in patients until 30 h post-multiple administration. Nineteen patients exhibited normal VWF-dependent aggregation of platelets within 48–168 h post first bolus injection (140). Later, in a Phase II trial (TITAN), 75 acquired TTP patients received caplacizumab (10 mg) and 39 received placebo intravenously, from 6 h to 15 min before initiation of plasma exchange. Thereafter, caplacizumab was administered subcutaneously in 10 mg doses daily within half an hour of the exchange. While the time of response (confirmed normalization of platelet count) was the primary end-point of the study, exacerbations and relapses formed major secondary end-points. The daily administration of the drug continued for 30 days following plasma exchange, reaching the maximum duration of 90 days. Impressively, the median response time with the drug decreased by 39% compared to placebo (median days 2.97 for caplacizumab vs. 4.79 for placebo, *p* = 0.005), along with an event rate ratio of 2.2 (95% CI 1.28–3.78, *p* −0.005) as compared to the placebo. While 11 patients in the placebo group had exacerbation, only three of the caplacizumab-treated patients showed the same. Within a month of cessation of the study drug, 8 patients had a relapse with baseline ADAMTS13 activity persistently below 10% in seven of them. This could mean that the underlying autoimmune disorder was not resolved completely (146). Mild to moderate, bleeding-related adverse events were reported at a higher frequency for the caplacizumab-treated group (54% cases) as compared to the placebo group (38% cases). However, the bleeding risk was not clinically-significant and the events did not require medical attention.

The results from this trial proved significant in building the case for caplacizumab in fast and sustained resolution of aTTP and paved the way for a phase III clinical trial.

This phase III study—the HERCULES trial—was performed on 145 patients (73 receiving placebo, 72 receiving caplacizumab) already receiving daily plasma exchange and corticosteroids. Duration to platelet count response formed the primary endpoint. Seventy-four percentage reduction (*p* < 0.00001) in the occurrence of composite primary endpoints was observed for patients on caplacizumab, with the incidence rate reducing to 12.7 vs. 49.3% for placebo-treated patients. Bleeding-related Treatment Emergent Adverse Events (TEAE) were 45.6% (*n* = 33) for caplacizumab in comparison to 23.3% (*n* = 17) for the placebo group. Following these remarkable results from the HERCULES trial, caplacizumab received its first global approval last year (2018) in the European Union for the treatment of adult aTTP patients, followed by approval from the US Food and Drug Administration (FDA) (136, 144).

In summary, VWF A1-GPIbα binding remains pre-eminent in targeting coronary heart diseases, owing to the elevated levels of VWF specifically in these conditions. Hence, impeding the capacity of VWF to recruit platelets at the injured arterial wall is a crucial factor in designing new anti-platelet regimens. Despite the substantial efforts to specifically target the VWF-GPIbα axis as described above, and notwithstanding the promising, preliminary preclinical and clinical results, no other inhibitors except ALX-0081 have received clinical approval so far.

Local shear-gradients have recently been recognized as the critical driving force behind pathological platelet aggregation, ultimately resulting in insidious thrombus formation. However, their paramount significance in thrombosis has been underplayed from the perspective of their exploitation for novel anti-thrombotic drug designs. Targeting the thrombosis-specific shear microgradients may prove to be the cornerstone in the field of antithrombotic therapeutics. While various direct anti-VWF agents are being developed (above), another approach is utilizing “smart” shear-responsive vehicles to locally deliver current anti-platelet drugs to sites of pathological thrombus formation, as we specify next.

### Shear-Sensitive Drug Delivery Platforms Targeting Occlusive Thrombi

**Figure 6:**
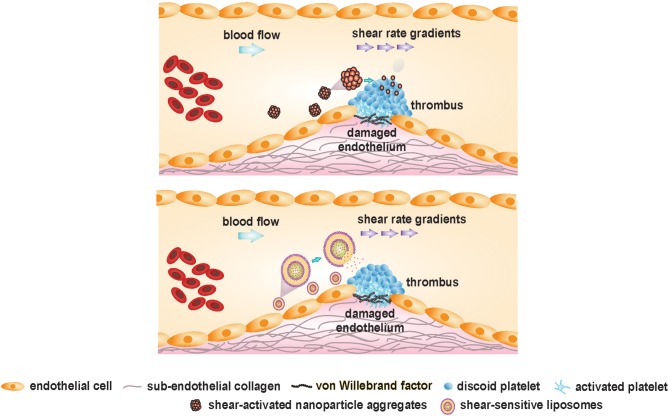
Shear-sensitive drug delivery platforms targeting occlusive thrombus formation. Adapted and modified from Westein et al. (151) with permission from the publisher.

Cardiovascular manifestations resulting from exacerbated thrombus formation are fundamentally distinct from physiological thrombus formation because of the abnormally high and complex blood shear forces associated with the latter. Exploitation of this spatially confined high shear environment could be achieved by delivering anti-thrombotic drugs using “smart,” shear-sensitive vehicles. Targeted delivery of current drugs, leveraging these unique shear forces, could improve the therapeutic out-turn of traditional anti-thrombotic drugs by resolving their associated bleeding limitations. Different shear-sensitive platforms have been outlined in Table 2 and are depicted in Figure 6. Those therapeutics, which have been reviewed elsewhere (147, 152–154), will be discussed briefly in this section.

**Table 2:** Outline of shear-sensitive drug delivery platforms targeting occluded blood vessels.

**Delivery platform**	**Carrier composition**	**Carrier morphology**	**Biologically active agent/loading method**	**Targeted site**	**Experimental setup**	**Experimental model**	**Experimental outcome**	**References**
**Nanoparticle aggregates**								
SA-NTs SA-NTs (combined with TEB)	PLGA PLGA	3.8 ± 1.6 μm aggregates composed of 180 ± 70 nm NPs 4 μm aggregates composed of ~200 nm NPs	tPA/coating r-tPA	Occluded mesentery artery Occluded pulmonary artery Occluded carotid artery	*In vivo* *In vivo* *In vivo*	Mouse arterial thrombus model Mouse PE model Rabbit carotid vessel occlusion model	Total dissolution of thrombus within 5 min of local administration Reinstatement of arterial flow with ~86% survival rate in mice post administration Higher rate of complete recanalization of ELVO with r-tPA coated SA-NTs with TEB, significantly less vascular trauma than stent-retriever TM	(147) (147) (148)
**Liposomes**								
Lenticular vesicles Nanocapsules	Pad-PC-Pad Egg-PC	Lenticular, 100 nm ~200 nm	[5(6)-FAM] Eptifibatide/encapsulation	– Occluded carotid artery	*In-vitro* (extracorporeal pump flow setup) *In-vivo*	– Mouse carotid vessel occlusion model	Selective payload (dye) release in stenosed arterial model at high shear Reduction of pulmonary thrombus load without prolonged bleeding times in mice	(149) (150)

*SA-NTs, shear-activated nanotherapeutics; SA-NPs, shear-activated nanoparticles; NPs, nanoparticles; PLGA, poly(lactic-co-glycolic acid); tPA, tissue plasminogen activator; PE, pulmonary embolism; TEB, temporary endovascular bypass; r-tPA, recombinant tissue plasminogen activator; ELVO, emergent large vessel occlusion; TM, thromboectomy; FAM, carboxyfluorescein (model-dye); Pad-PC-Pad, 1,3-dipalmitamidopropan-2-yl 2-(trimethylammonio)ethyl phosphate; PC, phosphatidylcholine*.

#### Shear-Activated Nanoparticle Aggregates

Korin and colleagues engineered micron-sized (1–5 μm), shear-activated nanotherapeutics (SA-NTs) or microscale aggregates from poly(lactic-co-glycolic acid), composed of smaller shear-activated nanoparticles (SA-NPs, ~200 nm) to target constricted, diseased blood vessels. The SA-NTs maintain a size similar to platelets, to circulate peripherally along the vessel wall, encountering minimal flow velocity and maximal shear rate (49).

The micro-sized aggregates are unique in their ability to breakdown only under pathologically-high shear rates, remaining intact under physiological shear. Consolidated via weak Van der Waals interactions among the constitutive nanoparticles, the aggregates disintegrate in the presence of overpowering shear forces at the pathological thrombus site. Following shear-triggered disintegration of the aggregates, disseminated nanoparticles (facing much lower hydrodynamic drag than the larger microaggregates) accumulate downstream of the stenosis, thereby releasing the cargo.

In the mesenteric ferric chloride-induced injury model, the intravenous administration of tissue-type plasminogen activator (t-PA)-coated SA-NPs resulted in rapid clot dissolution (within 5 min). Bolus injection of SA-NTs coated with 50 ng t-PA almost doubled the time to vessel occlusion vs. free, soluble t-PA at the same concentration. In a typically-fatal pulmonary embolism model in the mouse, almost 86% survival rate was observed in mice injected with t-PA-coated SA-NPs. Significantly reduced effective concentration of t-PA was indicated by the SA-NTs, that normalized pulmonary arterial pressure at a 100-times lower drug concentration than the control conditions (free drug). Targeted delivery of t-PA via SA-NPs, specifically to stenotic sites, can result in higher localized drug concentrations at stenotic sites without increased systemic concentrations of free drug, leading to lower hemorrhagic complications. So far, favorable results obtained from various *in vitro, ex vivo*, and *in vivo* models have illustrated the pre-clinical advantages of employing these engineered SA-NPs in achieving targeted delivery of the thrombolytic drug in a safe and effective manner. However, further investigations are essential to determine whether this targeting approach could be translated to the clinical setting. Furthermore, other clinically-approved antiplatelet and antithrombotic agents could also be loaded onto SA-NTs to prevent thrombosis.

#### Shear-Sensitive Liposomes or Vesicles

Liposomes are spherical vesicles (100–250 nm in diameter), consisting of an aqueous core surrounded by a phospholipid bilayer. Over the years, liposomal nanocapsules (NCs) have provided enormous opportunities in targeted drug delivery by their unique ability to load and stabilize a plethora of hydrophilic and hydrophobic drugs (155–158). In general, liposomes possess spherical morphology due to its minimum energy and maximum stability (159). This morphology imparts robustness to the vesicles against mechanical disruption. Bernard et al. demonstrated shear-induced membrane permeability in unilamellar, egg phosphatidyl choline (EPC) vesicles (50–400 nm) using a non-ionic surfactant, Brij 76. The study provided an experimental proof that on addition of detergents, membrane leakage can be induced by shear. Greater shear-driven membrane leakage was observed for large, unilamellar vesicles (LUVs) as compared to the smaller ones, in the presence of Brij 76 (160). Holme et al. fabricated non-spherical, lenticular vesicles (Pad-PC-Pad) exhibiting a relatively larger bending moduli and curvature around the equator, that could lead to transient pore formation under elevated shear stress. The lenticular morphology of these liposomes could increase their sensitivity toward higher shear levels. The liposomes were able to release the loaded fluorescent dye, carboxyfluorescein, under mechanical shaking even without the surfactant. However, no leakage of the liposomes was observed in the absence of mechanically disruptive forces. An extracorporeal circulation model was designed to mimic physiological and pathological (75% lumen restriction) arterial flow conditions with the shear stresses of 2 Pa and about 40 Pa, respectively. A clear, shear-specific fluorescent dye release was observed for the Pad-PC-Pad liposomes, with 45.5 ± 11% payload release in the healthy arteries and 70 ± 2.3% release in the constricted arteries after one passage only. In comparison, the fluorophore release by the egg PC liposomal formulations was below 10% after one passage. These promising *in vitro* results for the Pad-PC-Pad liposomes highlight their potential in the area of targeted, shear-specific delivery to the occluded vessels in MI patients (149).

Recently, Molloy et al. (150) formulated shear-sensitive egg phosphatidylcholine (PC) liposomes that targeted the clinical antiplatelet drug, eptifibatide, to the local thrombus site. *In vitro* blood perfusion assays were done in stenotic microchannels emulating stenotic shear conditions. The liposomes released eptifibatide at stenotic sites with governing shear rates of ~8,000 s^−1^, inhibiting platelet aggregation specifically in the narrowed region without effects on thrombus volume in regions of lower shear (~1,000 s^−1^). Modulation of the shear-sensitive drug release properties of the nanocapsules was demonstrated by incorporating Brij 76 in the formulations, reducing the liposomal shear-threshold from over 1,500 s^−1^ to between 500 and 1,000 s^−1^. When applied *in vivo* in a carotid artery occlusion mouse model, the shear-sensitive, eptifibatide-loaded liposomes significantly reduced thrombus load as compared with free eptifibatide, without any prolongation of bleeding times. This safety profile has most likely been achieved by the marked reduction in systemic eptifibatide concentrations, enabled by the targeted drug-release approach.

Surface functionalization of shear-sensitive liposomes with targeting moieties could actively direct the liposomes toward thrombi *in vivo*, further lowering the required dosage. Site-specific delivery of anti-platelet drugs could increase the local drug concentration at the stenosed vessel region without posing major bleeding risks.

In summary, liposomes can serve as an ideal carrier of an unprecedented range of antithrombotic agents, given the versatility of their physical and chemical structures and tunable functionality of the lipid surfaces to achieve desired physiological stimuli responsiveness. Tailoring their mechanical properties to induce sensitivity to pathological shear forces may result in efficacious, targeted drug delivery platform. Proof-of-concept studies have already shown promising results of liposomal drug delivery to constricted vessels with an increased local drug concentration. Drug dosage can be further reduced by chemical or biological modifications of liposomes via targeting moieties that direct the payload to the stenotic site, thereby attenuating thrombus progression (161–163).

## Conclusion and Current Clinical Challenges

VWF is an attractive, shear-sensitive target for drug delivery due to its altered biophysical properties under the pathological hemodynamic microenvironment, leading to hyper-aggregation of platelets in thrombotic events. Selective targeting of the VWF binding sites with platelet receptors could prove safer and more effective than systemic inhibition of platelet function via current antiplatelet drugs. Several agents targeting VWF under high shear conditions have been developed, yet despite initial success in some reports many challenges still require resolution for future clinical use. For example, *in vitro* assays and *in vivo* models emulating shear microenvironments in blood vessels adopt an over-simplified approach, while the physiological blood rheology and microvasculature vastly differ within and among organisms. This may account for the all too often failure to reproduce encouraging preclinical findings in clinical trials, seen with a number of compounds to-date.

Current blood coagulation tools, including tail bleeding time, skin puncture test, activated partial thromboplastin time (APTT), prothrombin time (PT) or platelet aggregometry do not incorporate biomechanical aspects of thrombosis, i.e., shear stress (164–166). The inability of the standard testing tools to embed the effects of local shear rates and shear rate gradients limits their assessment accuracy in the clinical settings. Rheological devices, such as parallel plate flow chambers and microfluidic blood perfusion channels coated with extracellular matrix proteins, can effectively emulate the pathological shear conditions in stenotic vessels. These devices provide versatile platforms with customizable flow and coating conditions, using minuscule blood volumes, to optimally indicate the net coagulative state of a given sample when all major pathways are in place (117, 120, 167, 168).

Several considerations should be taken in account when designing a novel shear-sensitive therapy, including the choice of a suitable, biologically-active compound(s), the need to tailor the shear sensitivity of a given template for maximum drug release without amplifying any negative effects, and the requirement to tune the circulation times (“half-life”) according to the drug's therapeutic window. This can be a challenging task, as most nanoparticles are immediately uptaken and rapidly cleared by the reticuloendothelial system (RES). Opsonization and immunogenicity can be other obstacles for *in vivo* utilization of these nano-agents (169). Therefore, optimization of particle hydrodynamic diameter, surface morphology and functionality can prolong their circulation times in blood to attain the desired therapeutic effect. PEGylation can also result in stealth nanoparticles with longer half-lives (169). Toxicity is yet another problem associated with nanoparticles, that can be significantly reduced or completely avoided by opting for natural compounds instead of synthetic ones (170). Storage stability as well as scale-up feasibility of nanoparticles can pose additional challenges in their production. Selecting a simple, cost-effective and easily reproducible production strategy can offer some remedy for these issues (171). Despite these challenges, nanoparticles are increasingly used in clinical applications (172).

## Future Perspectives

For decades, the focus of anti-platelet drug development has been on platelet activation pathways induced by soluble agonists and ligand binding (173, 174). In recent years, the concept of complex shear forces, which contribute to pathological thrombus formation, has gained traction in multiple studies using sophisticated mouse models (175, 176) as well as *in vitro* blood perfusion flow assays (4, 117, 120) and novel single-molecule techniques (7, 108, 177, 178). Nevertheless, so far these studies have not resulted in any translation into shear selective therapies.

The role of shear, and in particular gradients of shear, in driving VWF-GPIbα-dependent thrombus formation in cardiovascular diseases has become apparent. The difference in propensity of VWF-GPIbα interaction under constant, physiological shear rates and pathological shear gradients can be crucial in distinguishing between the life-saving process of hemostasis and the life-threatening process of thrombosis. Selective interference with the VWF-GPIbα axis under shear gradients may not only increase the efficacy of future anti-thrombotic agent, but potentially resolve the most dangerous limitation of current therapeutics, being bleeding complications.

Several strategies that selectively target VWF A1-GPIbα binding under high shear rates have been developed, including antibodies (AJvW-2, AJW200), aptamers (ARC1779, ARC15105), and nanobodies (ALX-0081). In a related strategy, shear-sensitive “smart” nanoparticles have been produced to target vessel occlusions, including nanoparticle aggregates and liposomes. Although further studies are required to validate the efficacy and safety of these therapeutic nanotechnologies, preliminary results have been highly promising.

Taken together, nanoparticles exhibit unique physical, chemical, structural, and biological properties, which can be harnessed to revolutionize the field of thrombosis. Nanoparticles versatility truly offers immense possibilities for surface modification and functionalization to suit the desired application. Nano formulations indeed provide great opportunities for targeted drug delivery due to their high drug payload capacity, reduced antigenicity and prolonged plasma half-life (179–182). Lower effective dosage and higher localization of the drug at the affected sites may prove critical in addressing the age-old bleeding issues. Although the full potential of shear-sensitive antithrombotic/antiplatelet therapy is yet to be clinically realized, shear-responsive strategies are destined to transform the future of thrombosis.

## Author Contributions

AR has drafted the first version of the paper and all other authors (CH, BN, and EW) have reviewed and revised the review.

### Conflict of Interest

The authors declare that the research was conducted in the absence of any commercial or financial relationships that could be construed as a potential conflict of interest.
